# Is HSD17B13 Genetic Variant a Protector for Liver Dysfunction? Future Perspective as a Potential Therapeutic Target

**DOI:** 10.3390/jpm11070619

**Published:** 2021-06-30

**Authors:** Takashi Motomura, Sriram Amirneni, Ricardo Diaz-Aragon, Lanuza A. P. Faccioli, Michelle R. Malizio, Michael C. Coard, Zehra N. Kocas-Kilicarslan, Carla Frau, Nils Haep, Alina Ostrowska, Rodrigo M. Florentino, Alejandro Soto-Gutierrez

**Affiliations:** 1 Department of Pathology, University of Pittsburgh School of Medicine, Pittsburgh, PA 15261, USA; motomurat1982@gmail.com (T.M.); sriramamirneni@gmail.com (S.A.); chambisdiaz@gmail.com (R.D.-A.); lanfaccioli@gmail.com (L.A.P.F.); mrm239@pitt.edu (M.R.M.); MCC143@pitt.edu (M.C.C.); zehranur.kocas@gmail.com (Z.N.K.-K.); carla.frau22@gmail.com (C.F.); nilshaep@gmail.com (N.H.); alina.ostrowska@chp.edu (A.O.); 2 Pittsburgh Liver Research Center, University of Pittsburgh, Pittsburgh, PA 15261, USA; 3 McGowan Institute for Regenerative Medicine, Pittsburgh, PA 15219, USA

**Keywords:** HSD17B13, fatty liver disease, NAFLD, iPS cell

## Abstract

As diet and lifestyle have changed, fatty liver disease (FLD) has become more and more prevalent. Many genetic risk factors, such as variants of PNPLA3, TM6SF2, GCKR, and MBOAT7, have previously been uncovered via genome wide association studies (GWAS) to be associated with FLD. In 2018, a genetic variant (rs72613567, T > TA) of hydroxysteroid 17-β dehydrogenase family 13 (HSD17B13) was first associated with a lower risk of developing alcoholic liver disease and non-alcoholic fatty liver disease (NAFLD) in minor allele carriers. Other HSD17B13 variants were also later linked with either lower inflammation scores among NAFLD patients or protection against NAFLD (rs6834314, A > G and rs9992651, G > A) respectively. HSD17B13 is a lipid droplet-associated protein, but its function is still ambiguous. Compared to the other genetic variants that increase risk for FLD, HSD17B13 variants serve a protective role, making this gene a potential therapeutic target. However, the mechanism by which these variants reduce the risk of developing FLD is still unclear. Because studies in cell lines and mouse models have produced conflicting results, human liver tissue modeling using induced pluripotent stem cells may be the best way to move forward and solve this mystery.

## 1. Introduction

Over the past few decades, liver diseases have mainly been induced by hepatitis viruses and hepatologists have been struggling to treat them [1]. Chronic infections caused by hepatitis B and C viruses have been linked to chronic hepatitis, fibrosis, and hepatocellular carcinoma (HCC), which has the second worse survival rate and is the fourth leading cause of cancer death worldwide [2,3]. Direct acting agents for hepatitis C, such as Ledipasvir/Sofosbuvir or Velpatasvir/Sofosbuvir [4,5] have enabled hepatologists to eradicate this pathogen. Reverse transcriptional agents, such as entecavir or tenofovir [6], as well as universal vaccination [7] seem to have promising therapeutic results for hepatitis B infection. However, in this half a century, another issue has developed along with lifestyle changes: fatty liver disease (FLD).

FLD has traditionally been classified as non-alcoholic fatty liver disease (NAFLD) and alcoholic liver disease (ALD) [8]. The concept of non-alcoholic steatohepatitis (NASH) first appeared in 1980, characterized by lobular inflammation and hepatocyte ballooning with lipid accumulation in the absence of severe alcohol intake [9]. Since then, growing global concern for NAFLD/NASH has coincided with the increase of obesity, type 2 diabetes, and the other metabolic syndromes [10,11]. Now, FLD has become the leading cause of chronic liver disease, affecting 25% of adults worldwide [12]. These patients are at the top of the list for liver transplantation [13]. In addition, FLD is the most common cause of HCC in Western countries [14]. Technical advances in genomic approaches have revealed that not only our lifestyle, but also genetic factors contribute to the risk of developing this disease.

For the first time, genome wide association studies (GWAS) described a missense variant (rs738409, C > G) in patatin-like phospholipase domain-containing 3 (PNPLA3), which was related to FLD [15]. This genetic variant encodes an isoleucine to methionine substitution at position 148aa (I148M) in the protein, which results in a loss of function of lipase activity of PNPLA3. This leads to lipid accumulation in the hepatocytes [16]. In the same GWAS study, another genetic variant was found in the transmembrane 6 super family member 2 (TM6SF2) gene [17]. The genetic variant (rs58542926, C > T) induces the replacement of glutamate with lysine at position 167aa (E167K), which also results in loss of function of TM6SF2. This leads to the reduction of secretion of very-low-density lipoprotein (VLDL) [18]. Another GWAS analyzing patients with hepatic steatosis, who had been diagnosed by CT imaging, revealed the association of a glucokinase regulator (GCKR) genetic variant (rs1260326, C > T) with hepatic steatosis [19]. This proline to leucine substitution (P446L) induces the loss of protein function, which increases de novo lipogenesis through glycolysis [20]. The membrane bound O-acyltransferase domain containing 7 (MBOAT7) genetic variant (rs641738, C > T) was first reported to be associated with alcoholic cirrhosis [21]. However, another European cohort study verified its correlation with NAFLD by histological examination [22]. The MBOAT7 genetic variant also causes a loss of protein function. This variant would no longer be able to acetylate lysophosphatidylinositol lipids, losing its protective role in preventing hepatic steatosis [23]. In another GWAS conducted in 2011, 42 loci were revealed to be associated with an increased concentration of liver enzymes, of which 10 were involved in lipid metabolism, such as PNPLA3 and GCKR [24].

Compared to these well-known genetic risk factors for FLD, the molecular mechanisms of hydroxysteroid 17-β dehydrogenase family 13 (HSD17B13), a genetic variant which was described in 2018 [25], have not been well uncovered yet. Nevertheless, this newly reported genetic variant, which is involved in various liver diseases, is now attracting broad attention from all four corners of the world. Interestingly, compared to the other genetic risks for FLD (Table 1), the HSD17B13 genetic variant (rs72613567, T > TA) is the only variant which plays a protective role, rather than being a risk factor. In this article, we review this poorly characterized genetic variant and discuss its possibility as a therapeutic target for liver diseases.

## 2. Molecular Function of HSD17B13

The HSD17B family has been described as containing 14 members until now, all of which display catalytic activity towards 17β-hydroxy and -keto steroid substrates [28]. These proteins are also known as members of the short chain alcohol reductase and dehydrogenase (SDR) protein superfamily [29], except for HSD17B5, which is an aldoketoreductase [30]. Interestingly, protein expression of HSD17B differs between human tissue, which suggests that HSD17B members have tissue-specific roles in local steroid metabolism [28].

Among members of HSD17B, the function of HSD17B13 is still unclear. The human HSD17B13 gene is located on chromosome 4 and contains 7 exons [27]. HSD17B13 was first isolated from a human adult liver cDNA library in 2007 and was originally named short chain dehydrogenase/reductase 9 (SCDR9) [31]. Shortly thereafter, Horiguchi et al. found that HSD17B13 was expressed dominantly in the liver and was a lipid droplet (LD) associated protein that localized around the LDs [32]. However, no other findings had been reported about this new liver specific lipid metabolic enzyme until 2014, when liquid chromatography mass spectrometry revealed the relationship between the expression level of HSD17B13 and LD accumulation in hepatocytes [33].

The HSD17B13 protein was found to be highly expressed in liver steatosis and in NAFLD liver samples by Su W et al. [33]. Additionally, in a mouse model with tail vein administration of adenovirus, HSD17B13 hepatic overexpression induced an increase in hepatic triglycerides. This result was corroborated by using hepatoma cell lines with plasmid transfection [33]. However, the correlation between simple hepatic steatosis and the HSD17B13 genetic variant was refuted by some clinical data [22,32]. On the other hand, Su W et al. reported that HSD17B13 expression was induced by liver X receptor alpha (LXRα), in a mouse model, and its transcription was nonexistent in sterol regulatory element binding protein (SREBP) 1c knock out mice [34]. Since both LXRα and SREBP-1c are key players in liver lipid metabolism, these results are an indication that HSD17B13 functions as a LD-associated protein, although the molecular mechanism remains unclear. [25,27].

## 3. Role of HSD17B13 Genetic Variants in Liver Disease

The correlation of HSD17B13 with FLD was first described in 2014 by Su W et al. [33]. Several years had passed before this association was in the spotlight again, when Abul-Husn et al. and Ma Y et al. revealed the correlation of HSD17B13 genetic variants with FLD [25,27]. Abul-Husn et al. [25] showed that rs72613567 T > TA, which is in a non-coding region between exon 6 and exon 7 of the HSD17B13 gene, is associated with low serum levels of alanine aminotransferase (ALT) and aspartate aminotransferase (AST). This GWAS was done by using 46,544 participants’ data from a cohort linked to the electronic health record. Ma Y et al. [27] described rs6834314 A > G, which is 11kb downstream of HSD17B13. This variant was significantly correlated with inflammation among 768 patients with NAFLD. In addition, rs6834314 has strong linkage disequilibrium with rs72613567 (D’ = 0.995, r^2^ = 0.93). Following these GWAS reports, many validation studies for the correlation of these genetic variants with various liver diseases have been conducted. Another group performed a genome-wide association study for abnormal liver function in the pediatric population, which also showed a correlation with an HSD17B13 genetic variant (rs3923441 C > G) [35].

### 3.1. Fatty Liver Disease

Abul-Husn et al. showed that the rs72613567 (T > TA) HSD17B13 genetic variant was originally found in association with low serum ALT levels [25]. However, this genetic variant minor allele (rs72613567:TA) was also significantly correlated with a 53% reduced risk for alcoholic liver disease and a 30% reduced risk for NAFLD among homozygotes [25]. In 2019, the rs6834314 minor allele (G) was also reported to reduce the inflammation score among NAFLD patients [27]. Recently, a European multinational group performed a GWAS using another 1483 pathologically-proven NAFLD cohort to detect the other genetic variant loci in the non-coding region of the HSD17B13 gene, rs9992651. This variant also has strong linkage disequilibrium with rs72613567, which has a protective effect against NAFLD with an odds ratio of 0.74 [26].

The genetic variants should be considered together with the different allele frequency among various races [36]. In addition, the incidence of FLD is related to the lifestyle in each country or region. According to the 1000 Genomes Project, the rs72613567 minor allele (TA) frequency is 18% [37], rising up to 34% in the East Asian population and 24% in the European population, whereas it is only 5% in the African population [36]. An Argentinian group confirmed that the rs72613567:TA variant prevented NAFLD and worse histologic outcomes (inflammation, ballooning, fibrosis) [38]. Kallwitz E et al. also showed that rs72613567:TA reduced the risk for suspected NAFLD and decreased the fibrosis score among the Hispanic/Latino population [39]. Regarding Asian countries, Seko Y et al. described that the HSD17B13 genetic variant attenuated the risk of the PNPLA3 minor allele for NAFLD [40]. Regardless of FLD, the protective effects of this variant on ALD or any chronic liver diseases were confirmed among the Chinese or Pakistani population [41,42].

With lifestyle changes around the world, FLD is not only a disease for adults anymore. FLD is now the leading cause of chronic liver disease among children, with a prevalence of 8% in the general pediatric population and of up to 34% in obese children [43]. The HSD17B13 genetic variant rs72613567 minor allele also reduced liver damage and even hepatic steatosis among obese children [44]. Based on these reports, we can conclude that the HSD17B13 genetic variant is linked to low risk for FLD (in both inflammation and fibrosis), regardless of the race, age, or etiology (alcoholic or non-alcoholic) of the patient.

### 3.2. Viral Hepatitis

Considering the function of HSD17B13 as a LD-associated protein, its association with FLD is clear. How about other chronic liver diseases, such as viral hepatitis? About F et al. reported the effect of the HSD17B13 genetic variant rs72613567 on HCV patients [45]. They enrolled 88 patients, including fibrotic and non-fibrotic patients, and the difference between the rs72613567 T/T and non-T/T was evident: the minor allele, TA, reduced the risk of developing HCV-associated fibrosis by 62% [45]. A European group published data with 3315 chronic liver disease patients and 33,337 healthy controls, in which TA allele carriers were significantly decreased among chronic hepatitis C patients compared to healthy controls (OR = 0.71, *p* = 0.0002) [46]. On the other hand, there was no correlation with hepatitis B patients. Enomoto H et al. investigated if the HSD17B13 genetic variants, rs72613567 and rs6834313, were correlated with the response rate for HBV treated with pegylated interferon therapy. No difference was found [47]. There is limited data to reach a conclusion on the relationship between HSD17B13 genetic variants and viral hepatitis. However, taking into consideration the fact that HCV needs and uses human liver lipid metabolism to form its infectious particles [48], there may be clues here to clarify the mechanism of HSD17B13 as a LD-associated protein.

### 3.3. Hepatocellular Carcinoma (HCC)

Chronic liver diseases can progress to serious conditions like cirrhosis and HCC. Yang J et al. first described the protective effect of the rs72613567 TA allele on the development of HCC among 3315 chronic liver disease patients. This protective effect was especially clear in ALD patients (OR = 0.64) [46]. Similar data was obtained from a 111,612 Danish population database (including 113 HCC) [49] and from a 6176 German cohort (including 1031 alcohol-related HCC) [50]. De Benedittis et al. reported that this genetic variant, rs72613567:TA, also attenuated the risk of HCC development among hepatitis C patients [51]. Because HCC arises more often in chronic liver disease patients with a higher fibrotic or inflammation score, it would not be surprising that the HSD17B13 genetic variant also prevents HCC development. However, according to De Benedittis et al., there are still differences in HCC occurrence, even among fibrotic patients carrying the T or the TA allele. This suggests that this genetic variant could also protect the liver directly from carcinogenesis.

Wang X et al. reported that HSD17B13 gene expression in the liver tissue could be used as a prognostic factor after hepatectomy for HCC. Low HSD17B13 expression led to worse survival [52]. Chen J et al. also reported similar results [53]. In addition, overexpressing HSD17B13 in the Huh-7 and SK-HEP-1 hepatoma cell lines led to an increase of cells in the G1 phase of the cell cycle, suggesting that HSD17B13 could potentially delay the cell cycle [53]. Furthermore, if this genetic variant truly lowers HSD17B13 enzymatic activity [25,27] and protects the liver, then further investigation is needed to uncover the role of HSD17B13 genetic variants in HCC carcinogenesis and recurrence.

### 3.4. Simple Steatosis

Whether the rs72613567:TA allele also protects from simple steatosis of the liver or causes lipid accumulation should be discussed. Abul-Husn et al. and Ma Y et al. showed that HSD17B13 rs72613567:TA and rs6834314:G produced a splicing variant protein which had less enzymatic activity and induced hepatic simple steatosis [25,27]. However, Su W et al. checked HSD17B13 overexpressed hepatoma cell lines and C57/B6 mouse and found an increase in lipogenesis [33]. The other European cohort performing a GWAS did not detect a correlation between the HSD17B13 genetic variant and hepatic steatosis [26]. Pirola C et al. reported the protective effect of rs72613567:TA against worse histologic outcome among NAFLD patients, but the grade of steatosis was not included in the report [38]. Although there was no statistical data, the authors showed that liver tissue from rs72613567:TA/TA homozygous carriers contain more LDs than that observed from T/T carriers.

## 4. Role of HSD17B13 Genetic Variants in the Other Diseases

Although HSD17B13 is expressed predominantly in the liver [32], it would not be surprising if this genetic variant plays a role in extrahepatic diseases, considering its enzymatic function in lipid metabolism. Ratroff D.M et al. performed a GWAS for type 2 diabetes (T2D) patients who were participants in a clinical trial comparing the benefits of fenofibrate for dislipidemia [54]. In white subjects, they found that the HSD17B13 genetic variant was associated with higher serum levels of triglycerides (TG) and high density-lipoprotein (HDL) [54]. Xu L et al. checked if the HSD17B13 genetic variant was associated with cardiovascular disease (CVD) to confirm the negative correlation of the rs6834314 minor allele with serum ALT levels among ischemic heart disease (IHD) and CVD patients, as well as with serum TG level [55]. None of the other factors, such as blood pressure, heart rate, QT interval and number of IHD, and T2D, were correlated. To date, there is no data to confirm that the HSD17B13 genetic variant directly protects from T2D or CVD, although it could have some sort of effect on improving these diseases through its LD-associated enzyme function. Despite limited data among obese children, Di Sessa A et al. reported that renal function (estimated glomerular filtration rate) was always worse at any age in those who carried the rs72613567 major homozygote allele than in those who carried the minor TA allele, at least under the age of 18 [56]. They hypothesized the function of HSD17B13 as a retinol metabolism enzyme that might be involved with renal protecting effect, but we have no supporting evidence yet.

The HSD17B13 genetic variant could protect from inflammation, fibrosis, and carcinogenesis caused by FLD and hepatitis C. It might have some potential to protect renal function and to decrease the severity of CVD, but further studies are needed to clarify its mechanism.

## 5. HSD17B13 as a Potential Therapeutic Target

Unfortunately, there are no specific pharmacotherapies for FLD currently. According to the latest guidance by the American Association for the Study of Liver Diseases (AASLD), only vitamin E, pioglitazone, ω3 fatty acid and statins are mentioned as the pharmacotherapies that “could be” or “may be” used as a treatment option for NASH/NAFLD [57]. Among these, pioglitazone, a ligand for the nuclear transcription factor peroxisome proliferator-activated receptor gamma (PPAR-γ), and vitamin E, an anti-oxidant agent, have been shown to improve steatosis, inflammation, and ballooning, which are the histological features of NASH/NAFLD [57]. These therapies have also been connected to some adverse effects, such as weight gain, prostate cancer, decreased bone mineral density, and bladder cancers. Because of this, the AASLD recommends using these treatments for biopsy-proven NASH/NAFLD after the discussion of benefits and risks with each patient [57]. Interestingly, bariatric surgery has been reported to ameliorate the histopathology of NASH/NAFLD patients in some retrospective or prospective cohort studies [58,59].

**Figure 1:**
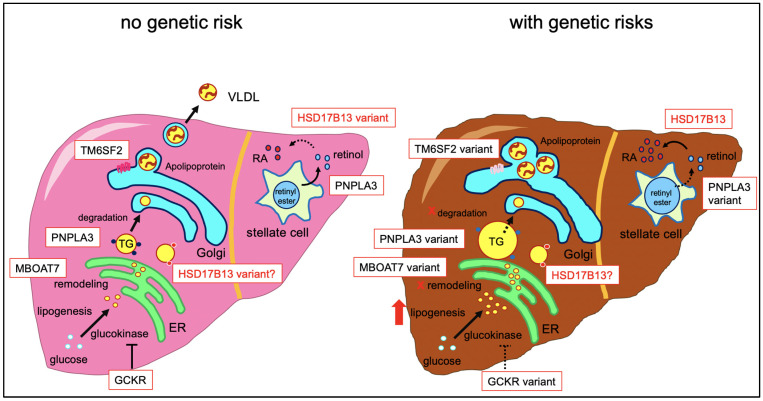
Molecular mechanism of genetic variants in FLD. HSD17B13 localizes at the surface of lipid droplets, but the function it serves there is still unknown. HSD17B13 also acts as a retinoic dehydrogenase, converting retinol into retinoic acid (RA). The HSD17B13 variants lose this function, which may protect the liver, but studies in stellate cells and their interactions with hepatocytes are still missing.

The demonstrated genomic risk factors for FLD, which the recent advances in genomic studies have uncovered, are expected to be potential therapeutic targets. However, considering the molecular mechanisms, targeting these genetic variants could be a “double-edged sword”. The GCKR genetic variant alters glucokinase activity that leads to the promotion of liver glucose metabolism and increases de novo lipogenesis in the liver [60]. In other words, recovering this enzymatic activity would improve hepatic steatosis, but also increase the fasting plasma glucose level, which may cause diabetes. The TM6SF2 genetic variant causes loss of function of secretion of lipoprotein from the liver [18]. Upregulation of this protein leads to the reduction of liver lipid accumulation but increases the circulating lipoprotein level and risk of developing CVD. Since PNPLA3 I148M reduces lipolysis and VLDL secretion in hepatocytes [61], therapeutic targeting of this genetic variant also has the chance to affect liver function and CVD at the same time, in opposite ways (Figure 1).

As mentioned above, the mechanism of how the HSD17B13 genetic variant protects the liver has yet to be clarified in detail. This genetic variant lowers serum TG and ALT levels among CVD patients [55] and could have some potential to improve renal function [56]. The HSD17B13 rs72613567 minor allele is the only genetic variant that is not a risk factor, but rather protects liver function. This could be the light at the end of the tunnel for seeking a therapeutic target for FLD.

How can we explore the therapeutic potential of the HSD17B13 genetic variant? One approach could be its RDH function. Ma Y et al. found reduced RDH activity in HSD17B13 minor allele carriers and hypothesized that the decrease in retinoic acid or retinol binding protein 4, due to lack of RDH activity, could modulate stellate cell fibrogenesis [27]. However, this effect was proven otherwise by the same group using HSD17B13 knock out (KO) mice [62]. Having eaten a high fat diet/Western diet, the HSD17B13 KO mice showed no differences in liver injury, measured by blood ALT levels, and fibrosis compared to the wild type animal. However, the authors did show a worsening of steatosis and an upregulation of inflammatory genes in the KO mice, compared to the wild type mice. Obviously, differences between mice and humans are expected, but caution should be taken when targeting RDH as a treatment option unless its mechanism is more clearly revealed.

Along with the advance of genomic studies, genome editing techniques have also tremendously developed. For example, back in 2014, a successful clinical trial for human immunodeficiency virus (HIV) treatment was published using autologous CD4+ T cells in which the C-C motif receptor 5 (CCR5) was modified by zinc-finger nuclease [63]. Additional methods for gene editing have been developed lately, such as transcriptional activator-like effector nuclease (TALEN) and CRISPR-associated nuclease, which are easier to engineer and can be applied more broadly to life sciences [64]. TALEN can recognize the specific sequence with greater accuracy than zinc-finger nuclease and CRISPR-associated nuclease can target the sequence with higher efficiency, making them powerful genome-editing tools [64]. Using these techniques, the HSD17B13 genotype could be genetically edited in hepatocytes. However, unlike peripheral blood cell transplantation, cell transplantation for solid organs, including the liver, is still extraordinarily limited for humans [65]. Genetic alterations in cell lines [27] or in mice [62] are possible and are relatively easy to perform, but with regard to the HSD17B13 genetic variant at least, the results show a discrepancy between the two models. It seems difficult to utilize hepatoma cells or mice as a model system to understand therapeutic targets for FLD. Therefore, a better model to study the HSD17B13 gene and its variants is needed, one that is both reliable and mimics the cellular processes that occur in humans.

Production of human hepatocytes or liver tissue without multicellular cues, from genome-edited induced pluripotent stem (iPS) cells, has been established [66,67]. Using these approaches, we can compare metabolomic differences between human normal hepatocytes and between livers that differ only in HSD17B13 genetic variants. iPS cells have the potential to settle the inconsistencies between human and non-human models when it comes to the role of the HSD17B13 variant. In addition, human iPS models allow for the creation of human derived, genome-edited hepatocytes that only vary in the genotype of HSD17B13. By controlling for all other genetic variations, investigators could study the direct effects on cellular processes that HSD17B13 variants could induce. Ma Y et al. hypothesized that the decreased retinol in hepatocytes or stellate cells is due to the loss of function of the RDH activity of HSD17B13 in minor allele carriers, which would protect the liver [27]. No previous study has been able to investigate the interactions between hepatocytes and stellate cells of the same genotype and genetic background. However, iPS cells and gene editing tools could make this possible, as both hepatocytes and stellate cells can be derived from a single iPS cell line. Since HSD17B13 is a novel gene, its various functions have yet to be clarified in detail. As a result, the molecular mechanism in which the genetic variant protects the liver is unknown. Unlike the other genetic variants for FLD, the HSD17B13 variant minor allele protects the liver and is associated with a lower risk of developing CVD. Studying the HSD17B13 genetic variant and discovering its mechanism using iPS cells and genetic engineering could be the best suited method to pave the way for personalized targeted therapy for FLD.

## 6. Conclusions

The protective role of these newly discovered genetic variants of HSD17B13 needs further study. Experiments conducted in knock-in/knock-out models of mice produced results that conflicted with those conducted in cell line models. Additionally, clinical samples have varying genetic and lifestyle backgrounds, making it difficult to compare between approaches. The HSD17B13 genetic variant minor allele can still be a good therapeutic target, protecting the liver and reducing the risk of developing CVD. The best model to investigate how this occurs may be iPS cells. Both hepatocytes and stellate cells can be generated from these iPS cells, making it possible to study the role of the HSD17B13 variant in both cell types. Furthermore, these cells have the potential to be gene edited and will contain the same genetic background. iPS cells could help uncover the protective mechanism of the HSD17B13 genetic variant and expose a therapeutic target for FLD, solving the biggest hurdle in liver disease of this era.

## Figures and Tables

**Table 1:** Genetic Variants Associated with FLD.

Gene	SNP	MAF	NAFLD Allelic OR (95% CI)	ALD Allelic OR (95% CI)	HCC Allelic OR (95% CI)	Assumed Molecular Mechanism of the Variant
PNPLA3	rs738409 (C > G)	0.14 (Africans)– 0.57 (Hispanics)	1.91 (1.64–2.21)	2.19 (1.97–2.43)	5.9 (1.5–23.8)	Repressor of lipase activity in hepatocyte [16] and altered retinol metabolism in stellate cell
TM6SF2	rs5842926 (G > A) rs58542926 (C > T) rs10401969 (T > C)	0.03 (Hispanics)– 0.08 (Europeans)	1.82 (1.59–2.08)	–	1.72 (1.27–2.38)	Loss of function of secretion of VLDL particle, leading lipid accumulation in the liver [18]
GCKR	rs1260326 (C > T) rs780094 (T > C)	0.5 (Asians)– 0.86 (Africans)	1.38 (1.25–1.53)	–	1.84 (1.23–2.75)	Loss of affinity for glucokinase, leading to increased lipogenesis [20]
MBOAT7	rs641738 (C > T)	0.24 (Asians)– 0.42 (Europeans)	1.42 (1.07–1.91)	1.35 (1.23–1.49)	2.10 (1.33–3.31)	Loss of remodeling of phosphatidylinositol, resulting in increased TG synthesis [23]
LEPR	rs12077210 (C > T)	0.01 (Asians)– 0.28 (Africans)	1.48 (1.29—1.71)	–	–	Loss of leptin receptor function [26]
HSD17B13	rs72613567 (T > TA) rs6834314 (A > G) rs9992651 (G > A) rs3923441 (C > G)	0.06 (Africans)– 0.34 (Asians)	0.70 (0.57–0.87)	0.47 (0.23–0.97)	0.72 (0.66–0.79)	Loss of retinol dehydrogenase activity [27]

ALD: alcoholic liver disease, HCC: hepatocellular carcinoma, MAF: minor allele frequency, NAFLD: non-alcoholic liver disease, OR: odds ratio, SNP: single nucleotide polymorphism, TG: triglyceride.

## Data Availability

Not applicable.

## References

[1] Meringer H., Shibolet O., Deutsch L. (2019). Hepatocellular carcinoma in the post-hepatitis C virus era: Should we change the paradigm?. World J. Gastroenterol..

[2] Chen L., Abou-Alfa G.K., Zheng B., Liu J.-F., Bai J., Du L.-T., Qian Y.-S., Fan R., Liu X.-L., Wu L. (2021). Genome-scale profiling of circulating cell-free DNA signatures for early detection of hepatocellular carcinoma in cirrhotic patients. Cell Res..

[3] Villanueva A. (2019). Hepatocellular Carcinoma. N. Engl. J. Med..

[4] European Association for the Study of the Liver (2018). EASL Recommendations on Treatment of Hepatitis C 2018. J. Hepatol..

[5] Belperio P.S., Shahoumian T.A., Loomis T.P., Mole L.A., Backus L.I. (2019). Real-world effectiveness of daclatasvir plus sofosbuvir and velpatasvir/sofosbuvir in hepatitis C genotype 2 and 3. J. Hepatol..

[6] Tseng C.-H., Hsu Y.-C., Chen T.-H., Ji F., Chen I.-S., Tsai Y.-N., Hai H., Thuy L.T.T., Hosaka T., Sezaki H. (2020). Hepatocellular carcinoma incidence with tenofovir versus entecavir in chronic hepatitis B: A systematic review and meta-analysis. Lancet Gastroenterol. Hepatol..

[7] Mirdad R.S., Hyer J.M., Diaz A., Tsilimigras D.I., Azap R.A., Paro A., Pawlik T.M. (2021). Postoperative imaging surveillance for hepatocellular carcinoma: How much is enough?. J. Surg. Oncol..

[8] Valenti L., Pelusi S. (2020). Redefining fatty liver disease classification in 2020. Liver Int..

[9] Ludwig J., Viggiano T.R., McGill D.B., Oh B.J. (1980). Nonalcoholic steatohepatitis: Mayo Clinic experiences with a hitherto unnamed disease. Mayo Clin. Proc..

[10] European Association for the Study of the Liver (EASL), European Association for the Study of Diabetes (EASD), European Association for the Study of Obesity (EASO) (2016). EASL-EASD-EASO Clinical Practice Guidelines for the management of non-alcoholic fatty liver disease. J. Hepatol..

[11] Valenti L., Bugianesi E., Pajvani U., Targher G. (2016). Nonalcoholic fatty liver disease: Cause or consequence of type 2 diabetes?. Liver Int..

[12] Younossi Z., Tacke F., Arrese M., Sharma B.C., Mostafa I., Bugianesi E., Wong V.W.-S., Yilmaz Y., George J., Fan J. (2019). Global Perspectives on Nonalcoholic Fatty Liver Disease and Nonalcoholic Steatohepatitis. Hepatology.

[13] Crespo G. (2021). Moving Forward in the Stratification of Cardiac Risk in Liver Transplantation Candidates. Liver Transplant..

[14] Yen Y.-H., Cheng Y.-F., Wang J.-H., Lin C.-C., Wang C.-C. (2021). Characteristics and etiologies of hepatocellular carcinoma in patients without cirrhosis: When East meets West. PLoS ONE.

[15] Romeo S., Kozlitina J., Xing C., Pertsemlidis A., Cox D., Pennacchio L.A., Boerwinkle E., Cohen J.C., Hobbs H.H. (2008). Genetic variation in PNPLA3 confers susceptibility to nonalcoholic fatty liver disease. Nat. Genet..

[16] Pingitore P., Pirazzi C., Mancina R.M., Motta B.M., Indiveri C., Pujia A., Montalcini T., Hedfalk K., Romeo S. (2014). Recombinant PNPLA3 protein shows triglyceride hydrolase activity and its I148M mutation results in loss of function. Biochim. Biophys. Acta Mol. Cell Biol. Lipids.

[17] Kozlitina J., Smagris E., Stender S., Nordestgaard B.G., Zhou H.H., Tybjærg-Hansen A., Vogt T.F., Hobbs H.H., Cohen J.C. (2014). Exome-wide association study identifies a TM6SF2 variant that confers susceptibility to nonalcoholic fatty liver disease. Nat. Genet..

[18] Prill S., Caddeo A., Baselli G., Jamialahmadi O., Dongiovanni P., Rametta R., Kanebratt K.P., Pujia A., Pingitore P., Mancina R.M. (2019). The TM6SF2 E167K genetic variant induces lipid biosynthesis and reduces apolipoprotein B secretion in human hepatic 3D spheroids. Sci. Rep..

[19] Speliotes E.K., Yerges-Armstrong L.M., Wu L., Hernaez R., Kim L.J., Palmer C.D., Gudnason V., Eiriksdottir G., Garcia M.E., Launer L.J. (2011). Genome-wide association analysis identifies variants associated with nonalcoholic fatty liver disease that have distinct effects on metabolic traits. PLoS Genet..

[20] Raimondo A., Rees M.G., Gloyn A.L. (2015). Glucokinase regulatory protein: Complexity at the crossroads of triglyceride and glucose metabolism. Curr. Opin. Lipidol..

[21] Buch S., Stickel F., Trépo E., Way M., Herrmann A., Nischalke H.D., Brosch M., Rosendahl J., Berg T., Ridinger M. (2015). A genome-wide association study confirms PNPLA3 and identifies TM6SF2 and MBOAT7 as risk loci for alcohol-related cirrhosis. Nat. Genet..

[22] Mancina R.M., Dongiovanni P., Petta S., Pingitore P., Meroni M., Rametta R., Borén J., Montalcini T., Pujia A., Wiklund O. (2016). The MBOAT7-TMC4 Variant rs641738 Increases Risk of Nonalcoholic Fatty Liver Disease in Individuals of European Descent. Gastroenterology.

[23] Thangapandi V.R., Knittelfelder O., Brosch M., Patsenker E., Vvedenskaya O., Buch S., Hinz S., Hendricks A., Nati M., Herrmann A. (2021). Loss of hepatic Mboat7 leads to liver fibrosis. Gut.

[24] Chambers J.C., Zhang W., Sehmi J., Li X., Wass M., Van Der Harst P., Holm H., Sanna S., Kavousi M., Alcohol Genome-wide Association (AlcGen) Consortium (2011). Genome-wide association study identifies loci influencing concentrations of liver enzymes in plasma. Nat. Genet..

[25] Abul-Husn N.S., Cheng X., Li A.H., Xin Y., Schurmann C., Stevis P., Liu Y., Kozlitina J., Stender S., Wood G.C. (2018). A Protein-TruncatingHSD17B13Variant and Protection from Chronic Liver Disease. New Engl. J. Med..

[26] Anstee Q.M., Darlay R., Cockell S., Meroni M., Govaere O., Tiniakos D., Burt A.D., Bedossa P., Palmer J., Liu Y.-L. (2020). Genome-wide association study of non-alcoholic fatty liver and steatohepatitis in a histologically characterised cohort. J. Hepatol..

[27] Ma Y., Belyaeva O.V., Brown P.M., Fujita K., Valles K., Karki S., De Boer Y.S., Koh C., Chen Y., Du X. (2019). 17-Beta Hydroxysteroid Dehydrogenase 13 Is a Hepatic Retinol Dehydrogenase Associated With Histological Features of Nonalcoholic Fatty Liver Disease. Hepatology.

[28] Hiltunen J.K., Kastaniotis A.J., Autio K.J., Jiang G., Chen Z., Glumoff T. (2019). 17B-hydroxysteroid dehydrogenases as acyl thioester metabolizing enzymes. Mol. Cell. Endocrinol..

[29] Moeller G., Adamski J. (2006). Multifunctionality of human 17β-hydroxysteroid dehydrogenases. Mol. Cell. Endocrinol..

[30] Matsuura K., Shiraishi H., Hara A., Sato K., Deyashiki Y., Ninomiya M., Sakai S. (1998). Identification of a Principal mRNA Species for Human 3α-Hydroxysteroid Dehydrogenase Isoform (AKR1C3) That Exhibits High Prostaglandin D2 11-Ketoreductase Activity. J. Biochem..

[31] Liu S., Huang C., Li D., Ren W., Zhang H., Qi M., Li X., Yu L. (2007). Molecular cloning and expression analysis of a new gene for short-chain dehydrogenase/reductase 9. Acta Biochim. Pol..

[32] Horiguchi Y., Araki M., Motojima K. (2008). 17beta-Hydroxysteroid dehydrogenase type 13 is a liver-specific lipid droplet-associated protein. Biochem. Biophys. Res. Commun..

[33] Su W., Wang Y., Jia X., Wu W., Li L., Tian X., Li S., Wang C., Xu H., Cao J. (2014). Comparative proteomic study reveals 17β-HSD13 as a pathogenic protein in nonalcoholic fatty liver disease. Proc. Natl. Acad. Sci. USA.

[34] Su W., Peng J., Li S., Dai Y.-B., Wang C.-J., Xu H., Gao M., Ruan X.-Z., Gustafsson J.-Å., Guan Y.-F. (2017). Liver X receptor α induces 17β-hydroxysteroid dehydrogenase-13 expression through SREBP-1c. Am. J. Physiol. Metab..

[35] Namjou B., Network T.E., Lingren T., Huang Y., Parameswaran S., Cobb B.L., Stanaway I.B., Connolly J.J., Mentch F.D., Benoit B. (2019). GWAS and enrichment analyses of non-alcoholic fatty liver disease identify new trait-associated genes and pathways across eMERGE Network. BMC Med..

[36] Kozlitina J. (2020). Genetic Risk Factors and Disease Modifiers of Nonalcoholic Steatohepatitis. Gastroenterol. Clin. N. Am..

[37] Carlsson B., Lindén D., Brolén G., Liljeblad M., Bjursell M., Romeo S., Loomba R. (2020). Review article: The emerging role of genetics in precision medicine for patients with non-alcoholic steatohepatitis. Aliment. Pharmacol. Ther..

[38] Pirola C.J., Garaycoechea M., Flichman D., Arrese M., Martino J.S., Gazzi C., Castaño G.O., Sookoian S. (2019). Splice variant rs72613567 prevents worst histologic outcomes in patients with nonalcoholic fatty liver disease. J. Lipid Res..

[39] Kallwitz E., Tayo B.O., Kuniholm M.H., Daviglus M., Zeng D., Isasi C.R., Cotler S.J. (2020). Association of HSD17B13 rs72613567:TA with non-alcoholic fatty liver disease in Hispanics/Latinos. Liver Int..

[40] Seko Y., Yamaguchi K., Tochiki N., Yano K., Takahashi A., Okishio S., Kataoka S., Okuda K., Umemura A., Moriguchi M. (2020). Attenuated effect of PNPLA3 on hepatic fibrosis by HSD17B13 in Japanese patients with non-alcoholic fatty liver disease. Liver Int..

[41] Chen H., Zhang Y., Guo T., Yang F., Mao Y., Li L., Liu C., Gao H., Jin Y., Che Y. (2020). Genetic variant rs72613567 ofHSD17B13gene reduces alcohol-related liver disease risk in Chinese Han population. Liver Int..

[42] Raja A.M., Ciociola E., Ahmad I.N., Dar F.S., Naqvi S.M.S., Moaeen-Ud-Din M., Raja G.K., Romeo S., Mancina R.M. (2020). Genetic Susceptibility to Chronic Liver Disease in Individuals from Pakistan. Int. J. Mol. Sci..

[43] Anderson E.L., Howe L.D., Jones H.E., Higgins J., Lawlor D.A., Fraser A. (2015). The Prevalence of Non-Alcoholic Fatty Liver Disease in Children and Adolescents: A Systematic Review and Meta-Analysis. PLoS ONE.

[44] Di Sessa A., Umano G.R., Cirillo G., Marzuillo P., Arienzo M.R., Pedullà M., del Giudice E.M. (2020). The rs72613567:TA Variant in the Hydroxysteroid 17-beta Dehydrogenase 13 Gene Reduces Liver Damage in Obese Children. J. Pediatr. Gastroenterol. Nutr..

[45] About F., Abel L., Cobat A. (2018). HCV-Associated Liver Fibrosis and HSD17B13. N. Engl. J. Med..

[46] Yang J., Trépo E., Nahon P., Cao Q., Moreno C., Letouzé E., Imbeaud S., Bayard Q., Gustot T., Deviere J. (2019). A 17-Beta-Hydroxysteroid Dehydrogenase 13 Variant Protects from Hepatocellular Carcinoma Development in Alcoholic Liver Disease. Hepatology.

[47] Enomoto H., Aizawa N., Hasegawa K., Ikeda N., Sakai Y., Yoh K., Takata R., Yuri Y., Kishino K., Shimono Y. (2020). Possible Relevance of PNPLA3 and TLL1 Gene Polymorphisms to the Efficacy of PEG-IFN Therapy for HBV-Infected Patients. Int. J. Mol. Sci..

[48] Fukuhara T., Wada M., Nakamura S., Ono C., Shiokawa M., Yamamoto S., Motomura T., Okamoto T., Okuzaki D., Yamamoto M. (2014). Amphipathic α-Helices in Apolipoproteins Are Crucial to the Formation of Infectious Hepatitis C Virus Particles. PLoS Pathog..

[49] Gellert-Kristensen H., Nordestgaard B.G., Tybjaerg-Hansen A., Stender S. (2020). High Risk of Fatty Liver Disease Amplifies the Alanine Transaminase-Lowering Effect of a HSD17B13 Variant. Hepatology.

[50] Stickel F., Lutz P., Buch S., Nischalke H.D., Silva I., Rausch V., Fischer J., Weiss K.H., Gotthardt D., Rosendahl J. (2020). Genetic Variation in HSD17B13 Reduces the Risk of Developing Cirrhosis and Hepatocellular Carcinoma in Alcohol Misusers. Hepatology.

[51] De Benedittis C., Bellan M., Crevola M., Boin E., Barbaglia M.N., Mallela V.R., Ravanini P., Ceriani E., Fangazio S., Sainaghi P.P. (2020). Interplay of PNPLA3 and HSD17B13 Variants in Modulating the Risk of Hepatocellular Carcinoma among Hepatitis C Patients. Gastroenterol. Res. Pract..

[52] Wang X., Liao X., Yang C., Huang K., Yu T., Yu L., Han C., Zhu G., Zeng X., Liu Z. (2019). Identification of prognostic biomarkers for patients with hepatocellular carcinoma after hepatectomy. Oncol. Rep..

[53] Chen J., Zhuo J.-Y., Yang F., Liu Z.-K., Zhou L., Xie H.-Y., Xu X., Zheng S.-S. (2018). 17-beta-hydroxysteroid dehydrogenase 13 inhibits the progression and recurrence of hepatocellular carcinoma. Hepatobiliary Pancreat. Dis. Int..

[54] Rotroff D., Pijut S.S., Marvel S.W., Jack J.R., Havener T., Pujol A., Schluter A., Graf G.A., Ginsberg H.N., Shah H.S. (2018). Genetic Variants in HSD17B3, SMAD3, and IPO11 Impact Circulating Lipids in Response to Fenofibrate in Individuals with Type 2 Diabetes. Clin. Pharmacol. Ther..

[55] Xu L., Jiang C.Q., Lam T.H., Zhang W.S., Zhu F., Jin Y.L., Thomas G.N., Cheng K.K., Schooling C.M. (2016). Mendelian randomization estimates of alanine aminotransferase with cardiovascular disease: Guangzhou Biobank Cohort study. Hum. Mol. Genet..

[56] Di Sessa A., Umano G.R., Cirillo G., Passaro A.P., Verde V., Cozzolino D., Guarino S., Marzuillo P., Del Giudice E.M. (2020). Pediatric non-alcoholic fatty liver disease and kidney function: Effect of HSD17B13 variant. World J. Gastroenterol..

[57] Chalasani N., Younossi Z., LaVine J.E., Charlton M., Cusi K., Rinella M., Harrison S.A., Brunt E.M., Sanyal A.J. (2018). The diagnosis and management of nonalcoholic fatty liver disease: Practice guidance from the American Association for the Study of Liver Diseases. Hepatology.

[58] Mathurin P., Hollebecque A., Arnalsteen L., Buob D., Leteurtre E., Caiazzo R., Pigeyre M., Verkindt H., Dharancy S., Louvet A. (2009). Prospective Study of the Long-Term Effects of Bariatric Surgery on Liver Injury in Patients without Advanced Disease. Gastroenterology.

[59] Lassailly G., Caiazzo R., Buob D., Pigeyre M., Verkindt H., Labreuche J., Raverdy V., Leteurtre E., Dharancy S., Louvet A. (2015). Bariatric Surgery Reduces Features of Nonalcoholic Steatohepatitis in Morbidly Obese Patients. Gastroenterology.

[60] Beer N.L., Tribble N.D., McCulloch L.J., Roos C., Johnson P.R., Orho-Melander M., Gloyn A.L. (2009). The P446L variant in GCKR associated with fasting plasma glucose and triglyceride levels exerts its effect through increased glucokinase activity in liver. Hum. Mol. Genet..

[61] Pirazzi C., Adiels M., Burza M.A., Mancina R.M., Levin M., Ståhlman M., Taskinen M.-R., Orho-Melander M., Perman J., Pujia A. (2012). Patatin-like phospholipase domain-containing 3 (PNPLA3) I148M (rs738409) affects hepatic VLDL secretion in humans and in vitro. J. Hepatol..

[62] Ma Y., Brown P.M., Lin D.D., Ma J., Feng D., Belyaeva O.V., Podszun M.C., Roszik J., Allen J.N., Umarova R. (2021). 17-Beta Hydroxysteroid Dehydrogenase 13 Deficiency Does Not Protect Mice from Obesogenic Diet Injury. Hepatology.

[63] Tebas P., Stein D., Tang W.W., Frank I., Wang S.Q., Lee G., Spratt S.K., Surosky R.T., Giedlin M.A., Nichol G. (2014). Gene Editing ofCCR5in Autologous CD4 T Cells of Persons Infected with HIV. N. Engl. J. Med..

[64] Zhang H.-X., Zhang Y., Yin H. (2019). Genome Editing with mRNA Encoding ZFN, TALEN, and Cas9. Mol. Ther..

[65] Soltys K.A., Setoyama K., Tafaleng E.N., Gutiérrez A.S., Fong J., Fukumitsu K., Nishikawa T., Nagaya M., Sada R., Haberman K. (2017). Host conditioning and rejection monitoring in hepatocyte transplantation in humans. J. Hepatol..

[66] Takeishi K., De L’Hortet A.C., Wang Y., Handa K., Guzman-Lepe J., Matsubara K., Morita K., Jang S., Haep N., Florentino R.M. (2020). Assembly and Function of a Bioengineered Human Liver for Transplantation Generated Solely from Induced Pluripotent Stem Cells. Cell Rep..

[67] Collin de l’Hortet A., Takeishi K., Guzman-Lepe J., Morita K., Achreja A., Popovic B., Wang Y., Handa K., Mittal A., Meurs N. (2019). Generation of Human Fatty Livers Using Custom-Engineered Induced Pluripotent Stem Cells with Modifiable SIRT1 Metabolism. Cell Metab..

